# Association between air pollution, altitudes, and overweight/obesity in China

**DOI:** 10.3389/fpubh.2025.1589201

**Published:** 2025-07-10

**Authors:** Yugen Wang, Muchun Yu, Yanyan Liu

**Affiliations:** Department of Philosophy, College of Humanities and Social Sciences, Xi'an Jiaotong University, Xi'an, China

**Keywords:** obesity, air pollution, altitudes, concentration-response relationships, China study

## Abstract

**Background:**

Air pollution and altitudes are important obesogenic environmental risks. No studies have examined the influence of the co-exposure of these two risks and Body Mass Index (BMI). We discuss the concentration–response (C–R) relationships and potential mechanisms between nine air pollution, altitudes, and BMI.

**Methods:**

Data from 38,617 individuals aged 18–90 years in the China Family Panel Survey were used. Nine air exposure variables–Particulate Matter 2.5 (PM_2.5_), Sulfur dioxide (SO_2_), Carbon monoxide (CO), Nitrogen dioxide (NO_2_), Ozone (O_3_), Black Carbon (BC), Methane (CH_4_), Ammonia (NH_3_), and Non–Methane Volatile Organic Compounds (NMVOCs)–and altitude grid data were generated through the combination of satellite remote sensing inversion data and nationally representative surveys. Bayesian kernel machine regression and the moderated chain–mediation model were employed to examine the C–R relationships and potential mechanisms.

**Results:**

Four air pollution–PM_2.5_, BC, NMVOCs, and CH_4_-were positively associated with OW/OB. A “negative–positive–negative correlation” pattern across low altitudes (τ30 to τ55, 73.77–403.87 m), medium altitudes (τ55 to τ75, 403.88–944.73 m), and high altitudes (τ75 to τ99, 944.74–2,610.72 m) was revealed for the correlation between altitudes and BMI. Altitudes negatively moderated the relationship between air pollution and BMI. A chain mediator, consisting of physical activity and sleep quality sequentially, partially mediated the association between air pollution and BMI.

**Conclusions:**

Co-exposure of air pollution and altitude had a complex influence on individual BMI. Maintaining a healthy environment is important for the joint prevention and control of obesity.

## 1 Introduction

Overweight/obesity (OW/OB) constitutes a systemic threat to global health. Estimates suggest that 3.4 million deaths per year are attributable to obesity worldwide, accounting for ~4% of total years of life lost (YLL) and disability–adjusted life years (DALYs) (1). China faces a particularly severe OW/OB epidemic, where the number of adults diagnosed with obesity has quadrupled, while the OW/OB population has doubled since 2000–2024 (2, 3). The recent study forecasts that the prevalence of OW/OB may reach 65.3% by 2030, with projected healthcare expenditures reaching 418 billion yuan–equivalent to 22% of total national health expenditures (4, 5). These findings underscore the urgent need to identify determinants of OW/OB for targeted intervention (3).

Obesogenic environmental risks have been widely studied, yet predominantly in isolation (6–9). Individuals, however, are simultaneously exposed to multiple factors that interactively influence health outcomes. In China, the co–occurrence of air pollution and altitude exposure merits focused investigation given their synergistic impacts on weight regulation and metabolic homeostasis (10–12).

China faces severe air pollution, with an annual PM_2.5_ average of 25 μg/m3, five-fold higher than the WHO guideline (5 μg/m3) (1). Meanwhile, China has the most diverse altitudes distribution, with it's distinctive “Three–Step Staircase Topography”, as first step of the plateau (altitudes > 4,000 m), the second step of central plains (altitudes from 1,000 to 4,000 m), and the third step of plain (altitudes < 1,000 m) (13). Both atmospheric pollution and altitude gradients perturb metabolic homeostasis while shaping socioeconomically obesogenic environments, collectively imposing substantial health burdens. Elucidating their joint associations with body mass index (BMI) thus emerges as a critical research priority.

However, the influence of these two key obesogenic environments on BMI remains contentious. Regarding altitudes, Merrill concluded that high altitudes contribute to weight loss, with a consistent negative association observed at 500–2,499 m (14). Pajuelo-Ramírez et al. (11) further demonstrated that this association was more pronounced at higher altitudes (>3 000 m). In contrast, Peng et al.'s (15) study suggested the opposite, finding that high altitudes were associated with adverse metabolic outcomes linked to weight gain. As for air pollution, some studies have reported a positive association between BMI and exposure to particulate matter, nitrogen oxides, and sulfur oxides (12, 16, 17). However, other studies propose that certain pollution could induce leptin resistance, suppress appetite, and reduce energy expenditure, ultimately promoting weight loss (10, 18). Additionally, no studies have examined the combined influences of co–exposure to these two factors on BMI.

The indirect relationship between air pollution, altitude, and obesity is also worth attention, particularly their mediation. Since both air pollution and altitude are significant risk factors for behavior changes that contribute to obesity, it is reasonable to consider behavioral factors as a strong mediator. We selected physical activity (PA) and sleep quality (SQ) as key mediating variables. Although diet may be a more crucial factor, it has already been extensively analyzed in existing research. PA and SQ are systematically influenced by altitude and air pollution, and as important downstream factors, they mediate the relationship between environmental risks and obesity.

Accordingly, this study employed Bayesian Kernel Machine Regression (BKMR) to assess the joint associations of 10 environmental exposures–including nine air pollution (PM_2.5_, SO_2_, CO, NO_2_, O_3_, BC, CH_4_, NH_3_, NMVOCs) and altitude–with BMI in Chinese adults. Three objectives were addressed:

1. Quantifying concentration–response (C–R) relationships between all exposures and BMI.
2. Evaluating altitude's moderating role in air pollution–BMI associations.
3. Investigating mediating influences of physical activity (PA) and sleep quality (SQ) on the association between air pollution and BMI.

## 2 Methods

### 2.1 Study participants

The China Family Panel Survey (CFPS) is a national longitudinal study designed to assess demographic characteristics and health status among Chinese residents. Analyses utilized cross–sectional data, as longitudinal prefecture–level tracking was geographically fragmented. The data employs a multistage stratified sampling strategy, representing China's adult population (18–90 years) and conducted biennially since 2010. Prefecture–specific identifiers enable linkage of individual responses to satellite–derived environmental exposures for analyzing the associations between environmental exposures and BMI. Data from 2016, 2018, and 2020 waves were included. We excluded respondents with missing information on anthropometric measurements, socio–economic characteristics, and geographic location, or with unreliable measurements (height ≤ 50 or ≥250 cm, weight ≤ 30 or ≥300 kg) (16, 17). Pregnant women and those with self–reported history of cancer were also excluded. The final analytic sample comprised 38,617 individuals across 126 prefectures. Ethical approval was obtained from Peking University's Ethics Review Board, with written informed consent provided by all participants.

### 2.2 Selected variables

#### 2.2.1 Air exposures

Air pollution exposure indicators were derived from NASA Terra satellite remote sensing data (2016–2020), utilizing aerosol optical depth measurements from the Moderate Resolution Imaging Spectroradiometer (MODIS) and Multi–angle Imaging Spectroradiometer (MISR) (https://ciesin.columbia.edu/data). Annual mean concentrations (μg/m3) of PM_2.5_, SO_2_, CO, NO_2_, O_3_, BC, CH_4_, NH_3_, and NMVOCs were extracted at 1 km and 0.01° spatial resolution. Gridded data were spatially aligned with CFPS prefecture boundaries via bilinear resampling. We selected a 1 km buffer resolution, balancing exposure misclassification risks (undersampling at 10 km) and computational feasibility [overfitting at 250 m; (34)], based on empirical evidence that 1 km approximates the average daily mobility range of Chinese adults. The air pollution exposure value refers to the annual mean exposure for each respondent in the given survey year.

#### 2.2.2 Altitudes

Altitude data were sourced from the Consultative Group on International Agricultural Research–Consortium for Spatial Information (CGIAR–CSI) platform (http://srtm.csi.cgiar.org/), providing a spatial resolution of 3 arc–s. Altitude values for CFPS participants were extracted based on residential prefectures, with altitudes ranging from 2 to 3,500 m.

#### 2.2.3 BMI

BMI was defined as the value of weight divided by the square of height [weight (kg)/height (m^2^)].

#### 2.2.4 Mediation

We chose PA and SQ as the mediation of the correlation between air pollution and BMI. As for the PA, the CFPS survey collected self–reported data from respondents regarding their frequency in PA. Respondents were given four categorical options: no physical activity, less than once per week, one to four times per week, and more than five times per week. These categories are subsequently assigned numerical values of 1, 2, 3, and 4, respectively, to quantify the intensity of PA as “none”, “ < 1 time per week”, “1–4 times per week”, “>5 times per week”. This categorization of physical activity was based on several established studies, which aimed to determine the correspondence of self-reported physical activity time information to physical activity intensity (6, 9, 19).

The SQ data was collected in the CFPS through the self–reported questionnaire, with four available options of none insomnia, 1–2 times insomnia per week, 3–4 times insomnia per week, 5–7 times insomnia per week. The above categories are subsequently assigned numerical values of 4, 3, 2, and 1, to denote “high sleep quality”, “medium sleep quality”, “low sleep quality”, and “extremely low sleep quality”. This categorization of SQ was based on Pan et al.'s (20) studies, which aimed to determine the correspondence of self-reported insomnia information and SQ.

#### 2.2.5 Covariates

Demographic covariates were adjusted in this study, including age (years), sex (female, male), ethnicity (minority ethnicity, majority ethnicity), personal income (Chinese Yuan), education (illiterate, primary to middle, college), registration (rural, urban), marital status (married, unmarried), tobacco use (yes or no), alcohol consumption (yes or no).

### 2.3 Statistical analysis

First, cross–sectional univariate descriptive statistics of the main outcomes were presented by full subjects, OW/OB subjects, and normal weight subjects.

Second, we used the BKMR to capture the C–R relationships of each exposure on OW/OB. The BKMR model, a novel semi–parametric modeling approach, flexibly captured the joint association of the mixture components, allowing for potential interactions and non–linear associations. BKMR offered two appealing advantages compared to previous purely parametric or non–parametric approaches. First, it handled the joint association of multiple exposures using a kernel machine regression model, thereby capturing the potentially complex and non–linear joint C–R curves of multiple exposures while maintaining good statistical power. Second, it allowed for the disentangling of the joint association of mixtures into their main effects and moderation, while properly accounting for model uncertainty (21). The function of the BKMR model was:


$$\begin{array}{l} Y_{i}\;=\;h\left[Group\;=\;\left(\overset{c}{\underset{i=1}{\sum }}X_{i}\right)\right]+\beta^{T}Z_{i}+e_{i} \end{array}$$

Where *h*() was the C–R relationships of exposures on OW/OB, *Z*_*i*_ and β represented covariates and the coefficients. In our BKMR analysis, the number of iterations (iter) was set to 10,000, and the Gaussian Process Regression model was chosen.

**Figure 1:**
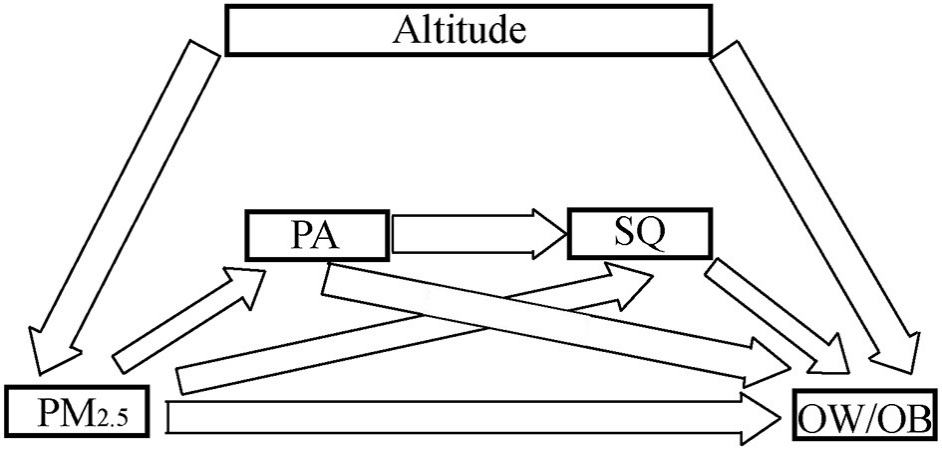
The potential mechanism between air pollution, PA, SQ, altitudes and BMI, which took the air pollution and BMI as the dependent and independent variable, PA and SQ as the sequential chain mediator, and altitudes as the moderator. Modification index and bootstraps (5,000 times) are used to optimize the model.

Third, the potential mechanism between air pollution, PA, SQ, altitudes and BMI was revealed by a moderated chain–mediation model, which took the air pollution and BMI as the dependent and independent variable, PA and SQ as the sequential chain mediator, and altitudes as the moderator, as shown in Figure 1. Modification index and bootstraps (5,000 times) were used to optimize the model.

The BKMR model and moderated chain–mediation model are conducted by the R (4.1.0) and Mplus (8.3).

## 3 Results

### 3.1 Population characteristics

The prevalence of OW/OB among Chinese adults was 46.86% between 2016 and 2020. Individuals with OW/OB tended to experience higher levels of air pollution, and lived at lower altitudes (Table 1).

**Table 1:** Characteristics of study participants in the Chinese Family Panel Surveys (CFPS), *N* = 38,617, 2016–2020.^af^

**Variables**	**All subjects (38,617)**	**OW/OB subjects^b^ (14,172)**	**Normal weight subjects^c^ (24,445)**	**χ^2^/*t*^d^**
BMI	23.04 (0.02)	26.71 (0.02)	20.91 (0.01)	< 0.01
${\text{PM}}_{2.5}^{\text{e}}$	3.86 (< 0.01)	3.89 (< 0.01)	3.84 (< 0.01)	< 0.01
${\text{SO}}_{2}^{\text{e}}$	3.26 (0.01)	3.26 (0.01)	3.26 (0.01)	0.02
CO^e^	0.55 (< 0.01)	0.57 (< 0.01)	0.55 (< 0.01)	0.03
${\text{NO}}_{2}^{\text{e}}$	3.65 (< 0.01)	3.67 (< 0.01)	3.63 (< 0.01)	0.02
${\text{O}}_{3}^{\text{e}}$	4.49 (< 0.01)	4.50 (0.01)	4.48 (0.01)	0.04
BC^e^	5.61 (< 0.01)	5.65 (< 0.01)	5.58 (< 0.01)	< 0.01
${\text{CH}}_{4}^{\text{e}}$	7.66 (0.01)	7.64 (0.01)	7.67 (< 0.01)	< 0.01
${\text{NH}}_{3}^{\text{e}}$	7.26 (< 0.01)	7.29 (0.01)	7.24 (< 0.01)	< 0.01
NMVOCs^e^	5.19 (< 0.01)	5.20 (< 0.01)	5.18 (< 0.01)	< 0.01
altitudes^e^	6.61 (0.03)	6.51 (< 0.01)	6.67 (< 0.01)	< 0.01
Male	0.49 (< 0.01)	0.52 (< 0.01)	0.47 (< 0.01)	< 0.01
Age (years)	45.81 (0.09)	48.06 (0.12)	44.52 (0.11)	< 0.01
18–40	41.32%	34.15%	45.48%	
41–60	26.21%	43.78%	31.83%	
61–90	22.46%	22.07%	22.69%	
Minority ethnicity	0.15 (0.01)	0.12 (0.02)	0.17 (0.01)	< 0.01
Personal income (Chinese Yuan)	37,164.7 (280.3)	39,220.6 (471.6)	35,931.23 (347.35)	0.03
≤ 10,000	72.03%	70.89%	72.69%	
10,001–50,000	19.95%	19.99%	19.93%	
50,001–100,000	7.09%	7.98%	6.57%	
≥100,001	0.93%	1.14%	0.81%	
Rural registration	0.25 (< 0.01)	0.28 (< 0.01)	0.23 (< 0.01)	< 0.01
Tobacco using	0.28 (< 0.01)	0.29 (< 0.01)	0.28 (< 0.01)	< 0.01
Alcohol using	0.14 (< 0.01)	0.15 (< 0.01)	0.13 (< 0.01)	< 0.01
**Physical activity**				
None	57.79%	55.90%	58.88%	0.02
< 1 time per week	7.69%	7.05%	8.07%	
1–4 times per week	10.50%	10.17%	10.69%	
>5 times per week	24.01%	26.87%	22.36%	
**Sleep quality**				
High sleep quality	7.70%	7.61%	7.75%	< 0.01
Medium sleep quality	12.95%	12.61%	13.14%	
Low sleep quality	31.51%	30.78%	31.94%	
Extremely low sleep quality	47.85%	49.01%	47.17%	

^a^Mean (standard deviation) for continuous variables, percentage for categorical variables.

^b^Restricting the sample of BMI ≥ 24.

^c^Restricting the sample of BMI < 24.

^d^χ^2^ test was used to identify the inter–group heterogeneity of categorical variables, and t-test was use to identify the inter–group heterogeneity of continuous variables.

^e^We take the logarithm of the exposures concentration.

^f^PM_2.5_ was Particulate Matter 2.5, SO_2_ was Sulfur dioxide, CO was Carbon monoxide, NO_2_ was Nitrogen dioxide, O_3_ was Ozone, BC was Black Carbon, CH_4_ was Methane, NH_3_ was Ammonia, NMVOCs was Non–Methane Volatile Organic Compounds.

### 3.2 The C–R relationships of air pollution and altitudes on OW/OB

**Figure 2:**
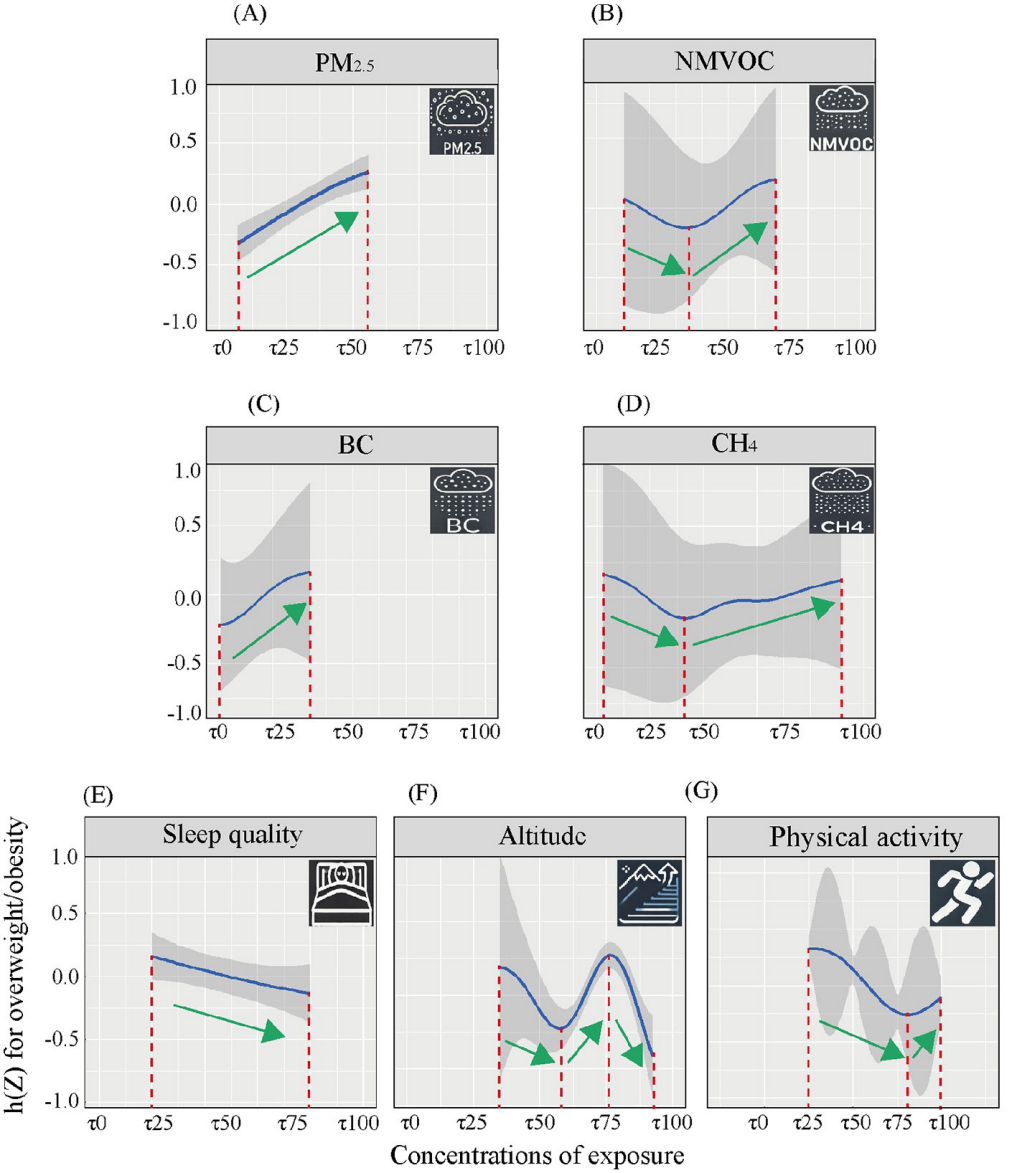
The C–R relationships between selected exposures and BMI estimated by BKMR model. This figure showed the relationship between specific exposure concentration and the individual BMI, when other exposures were kept at the median. **(A)** PM_2.5_ and OW/OB. **(B)** NMVOC and OW/OB. **(C)** BC and OW/OB. **(D)** CH_4_ and OW/OB. **(E)** Sleep quality and OW/OB. **(F)** Altitude and OW/OB. **(G)** Physical activity and OW/OB.

We first fitted the BKMR model to assess the joint association of mixed exposures with BMI. The selected exposures suffered from multicollinearity problems, with several variables had Variance Inflation Factors (VIF) > 10, see Table 2. The significant concentration–response (C–R) relationships of 7 exposures are shown in Figure 2. Four air pollution were significantly related to BMI: PM_2.5_ [positively associated with BMI at low–to–medium concentration tertiles (τ5–τ50)], BC [positively associated at low concentration tertiles (τ0–τ30)], NMVOC [negatively associated at low concentration tertiles (τ10–τ40) and positively associated at medium–to–high tertiles (τ40–τ75)], and CH_4_ [negatively associated at low concentration tertiles(τ0–τ40) and positively associated at medium–to–high tertiles (τ40–τ90)].

**Table 2:** Variance inflation factors test of multivariable in analysis.^a^

**Var**	**VIF**	**1/VIF**
PM_2.5_	8.86	0.11
SO_2_	9.47	0.11
CO	9.34	0.11
NO_2_	8.90	0.11
O_3_	9.09	0.11
BC	10.13	0.10
CH_4_	8.69	0.12
NH_3_	8.73	0.11
NMVOC	10.27	0.10
Altitudes	8.60	0.12
Cons	(-)	(-)

^a^Variance Inflation Factors >10 for the selected variable in the model indicates that there was a multicollinearity problem with this variable.

Altitudes was negatively associated with BMI at low concentration tertiles (τ30–τ55) and high concentration tertiles (τ75–τ100), and positively associated at medium concentration tertiles (τ55–τ75).

### 3.3 The potential mechanism of air pollution and altitudes on OW/OB

**Figure 3:**
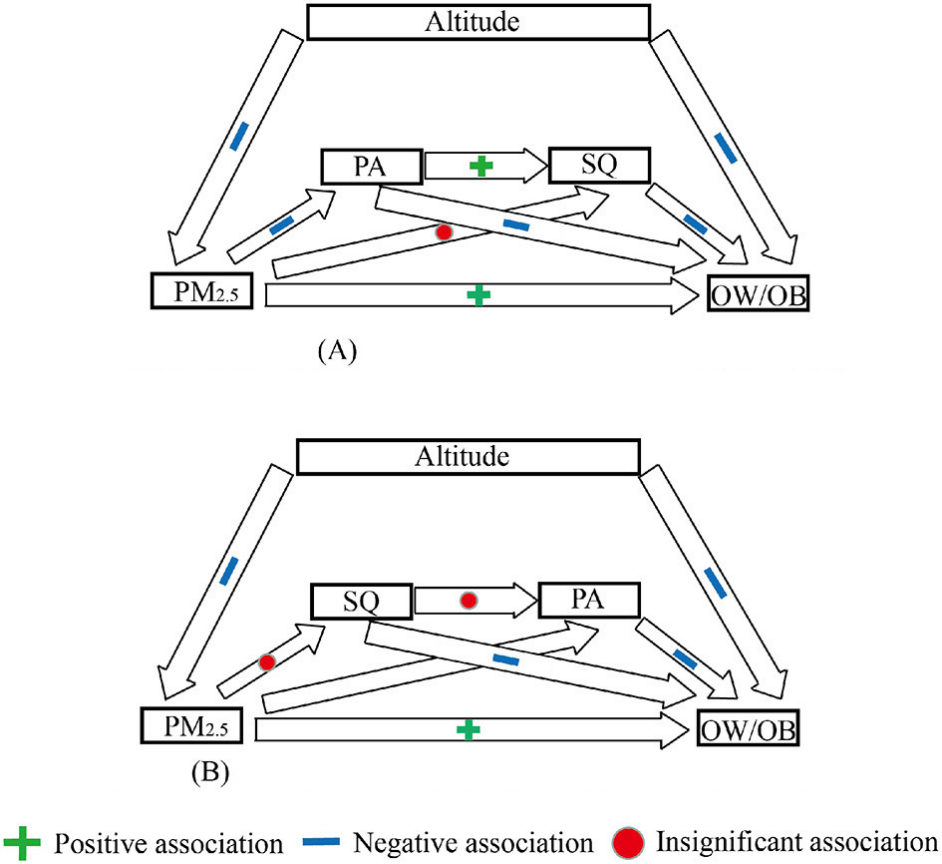
Potential association between PM_2.5_ and OW/OB: chain–mediated models. **(A)** Chain mediation model (CFI = 0.92 > 0.90; TLI = 0.96 > 0.90; SRMR = 0.041 < 0.08). **(B)** Chain mediation model for sensitivity test (CFI = 0.74 < 0.90; TLI = 0.82 < 0.90; SRMR = 0.069 < 0.08).

Table 3 and Figure 3 revealed the potential mechanisms between air pollution, PA, SQ, altitude, and obesity. For easy interpretation, we chose PM_2.5_ (with the most stable influence on BMI) as a proxy for air pollution. The chain mediation involving PA and SQ partially mediated the association between air pollution and OW/OB, accounting for 37.5% of the association (OR = 1.38, 95%CI = 1.27;1.51). Altitude negatively moderated the association between air pollution and BMI. A sensitivity analysis with a chain mediation model of “air pollution—SQ—PA—BMI” (Figure 3B) showed that reversing the order of SQ and PA reduced the model fit and rendered the mediation insignificant, indicating minimal impact on the results and confirming the robustness of our conclusions.

**Table 3:** Associations between exposures and overweight/obesity in SEM model (*N* = 38,617), CFPS, China, 2016–2020.^ab^

**Association**	**OR**	**95%CI**	***P*–value**
**Panel 1. Potential mediated association between PM**_**2.5**_ **and OW/OB (CFI** **=** **0.92** **>** **0.90; TLI** **=** **0.96** **>** **0.90; SRMR** **=** **0.041** **<** **0.08)**			
(a1) PM_2.5_ → Physical activity	1.31	1.19; 1.43	< 0.05
(a2) PM_2.5_ → Sleep quality	1.14	0.94; 1.38	>0.05
(b1) Physical activity → OW/OB	1.34	1.16; 1.52	< 0.05
(b2) Sleep quality → OW/OB	1.26	1.14; 1.41	< 0.05
(d) Physical activity → Sleep quality	1.17	1.08; 1.26	< 0.05
(c) PM_2.5_ → OW/OB	1.22	1.04; 1.43	< 0.05
Total	1.38	1.27; 1.51	< 0.05
Ind. total	1.13	1.06; 1.21	< 0.05
Ind1 (a1 × b1)	1.08	1.03; 1.14	< 0.05
Ind2 (a2 × b2)	1.03	1.01; 1.05	< 0.05
Ind3 (a1 × d × b2)	1.01	1.01; 1.02	< 0.05
**Panel 2. Potential moderation association between PM**_**2.5**_ **and OW/OB**			
(M) altitudes → PM_2.5_ and OW/OB (τ0–τ100)	0.85	0.73; 0.97	< 0.05
(M1) altitudes → PM_2.5_ and OW/OB (τ0–τ25)	0.57	0.48; 0.68	< 0.05
(M2) altitudes → PM_2.5_ and OW/OB (τ25–τ75)	1.45	1.15; 1.86	< 0.05
(M3) altitudes → PM_2.5_ and OW/OB (τ75–τ100)	0.66	0.55; 0.78	< 0.05

^a^All model adjusted for socio–economic variables [including age (years), sex (female, male), ethnicity (minority ethnicity, majority ethnicity), personal income (Chinese Yuan), education (illiterate, primary to middle, college), registration (rural, urban), marital status (married, unmarried)], and physical characteristics variables (including tobacco use (yes or no), alcohol consumption (yes or no).

^b^PM_2.5_ was Particulate Matter 2.5, SO_2_ was Sulfur dioxide, CO was Carbon monoxide, NO_2_ was Nitrogen dioxide, O_3_ was Ozone, BC was Black Carbon, CH_4_ was Methane, NH_3_ was Ammonia, NMVOC was Non–Methane Volatile Organic Compounds, PH was Pondus Hydrogenii value, NTU was Nephelometric Turbidity Units, KMnO4 was Potassium permanganate, P was Phosphorus, N was Nitrogen.

## 4 Discussion

We investigated the relationship between air pollution, altitudes, and individual BMI in 38,617 Chinese residents aged 18–90 years. Using the BKMR model, we established C–R relationships between 10 exposures and BMI, identifying positive associations for four air pollution (PM_2.5_, BC, NMVOC, and CH_4_) with BMI. A nonlinear C-R relationship was observed between altitudes and BMI, showing negative correlations at low altitude tertiles (τ30–τ55, 73.77–403.87 m), positive correlations at medium tertiles (τ55–τ75, 403.88–944.73 m), and negative correlations at high tertiles (τ75–τ99, 944.74–2,610.72 m). Additionally, altitudes negatively moderated the positive association between air pollution and BMI. Furthermore, a chain mediation involving PA and SQ partially mediated the association between air pollution and BMI.

### 4.1 Air pollution on OW/OB

An important objective of this study was to identify the detailed C–R association between mixed co–exposure to air pollution and altitudes on BMI. We found that four air pollutants–PM_2.5_, BC, NMVOC, and CH_4_-were positively associated with BMI. The results for PM_2.5_ and BC were consistent with previous studies. Bowe et al. demonstrated that a 10 μg/m3 annual increase in PM_2.5_ was linked to higher BMI (0.140 kg/m^2^ per year) and weight gain (0.968 pounds per year). This finding supports the hypothesis that long-term exposure to particulate matter, specifically PM_2.5_, contributes to an increase in body fat accumulation, likely through mechanisms such as inflammation, oxidative stress, and altered metabolic processes (21). Similarly, Friedman found that higher BC exposure was associated with increased fat mass percentage and fat mass index (22). Our study extended this understanding by revealing positive associations between PM_2.5_ and BC with BMI across concentration tertiles.

PM_2.5_ and BC were the only two air pollution factors that maintained stable positive associations with BMI. Notably, our findings show that both PM2.5 and BC maintained stable positive associations with BMI even at lower exposure levels, suggesting that these pollutants may have a threshold effect, where even moderate exposure can lead to adverse metabolic outcomes (16). This is important because it suggests that reducing air pollution, even by small increments, could potentially reduce obesity risk in populations exposed to these pollutants (10).

NMVOC and CH_4_ were positively associated with BMI only at the high–concentration tertile. This suggests that these pollutants may have a dose-dependent effect on BMI, where the impact becomes significant only after reaching certain exposure levels. No previous studies have focused on the influence of these two hazardous air pollutants on individual BMI. This gap in the literature highlights the novelty of our findings, which suggest that NMVOC and CH_4_ should be considered important contributors to obesity risk, particularly in regions with high pollution levels.

Laboratory evidence has shown that NMVOC exposure is linked to inflammation and oxidative stress, while excessive CH_4_ intake interferes with hormones, causing insulin resistance (23, 24). Both factors disrupt normal metabolic processes, increasing the risk of obesity. These disruptions may occur through complex pathways involving changes in hormone regulation, inflammatory responses, and fat cell metabolism. Notably, NMVOC and CH_4_ are byproducts of fossil fuel combustion, making them particularly relevant in countries like China, where industrial and transportation sectors contribute significantly to air pollution. The widespread use of fossil fuels in China poses a critical public health challenge, as these pollutants could exacerbate the growing obesity epidemic.

### 4.2 Altitudes on OW/OB

We identified a comprehensive C–R relationship between altitudes and BMI, characterized by a “negative correlation–positive correlation–negative correlation” pattern across low altitude tertiles (τ30–τ55, 73.77–403.87 m), medium altitude tertiles (τ55–τ75, 403.88–944.73 m), and high altitude tertiles (τ75–τ99, 944.74–2,610.72 m). In low and high altitude tertiles, altitudes were positively associated with BMI, whereas in medium altitude tertiles, altitudes were negatively associated with BMI.

In China, low-altitude regions and high-altitude regions commonly experience lower levels of economic development. This socioeconomic context compels local residents to rely heavily on labor-intensive occupations (e.g., agriculture, construction, mining) (25), resulting in chronic exposure to altitude-related hypoxic microenvironments (26). Although the absolute oxygen concentrations differ significantly between these two types of regions, the combination of occupational exposure patterns and hypoxia-mediated metabolic adaptations collectively drives the negative correlation between altitude and BMI.

In medium altitude regions of China, a positive correlation exists between altitude and BMI. This association stems from the geographic concentration of low-altitude zones in eastern coastal areas, where minimal elevations predominantly correspond to topographically constrained basins, while marginally higher elevations align with plains exhibiting superior economic development conditions (27). Enhanced economic status in these plains promotes obesogenic socioenvironmental drivers—including increased availability of energy-dense foods and reduced physical activity demands—thereby establishing a positive C-R relationship with BMI. Although altitude may theoretically influence metabolic processes through hypoxia-mediated pathways (e.g., oxygen availability reduction) (28), socioeconomic factors constitute the dominant explanatory mechanism for the observed altitude-BMI association in China's medium-altitude regions.

Altitudes negatively moderated the relationship between air pollution and BMI. This suggests that higher altitudes may buffer or reduce the harmful effects of air pollution on obesity risk. Mechanism analyses found that the higher the altitudes, the lower the influence of air pollution on individual BMI. This study revealed for the first time that altitudes mitigate the weight gain caused by air pollution, and three explanatory pathways might be identified: (1) air pollution was diluted in high-altitude regions, reducing individual exposure to hazardous pollution; At higher altitudes, lower air pressure and thinner air could contribute to the dispersal of pollutants, leading to reduced concentrations of harmful particles (29); (2) residents in high-altitude regions were typically more engaged in outdoor activity, such as walking and hiking, which counteracted the positive influence of air pollution on BMI; This is particularly relevant as higher physical activity levels can offset the weight gain associated with pollution exposure (19); and (3) high-altitude residents experienced physiological adaptations, such as increased erythropoiesis, improved oxygen efficiency, and enhanced sensitivity to insulin (28). These adaptations may enhance metabolic health, leading to better regulation of weight despite the presence of pollution. Such physiological mechanisms could improve overall fitness and promote weight loss, even in areas with significant air pollution.

### 4.3 Potential mechanism

A significant moderating effect of altitude was observed on the relationship between air pollution and overweight/obesity (OW/OB), indicating that the adverse health impact of air pollution on BMI diminishes at higher altitudes. This attenuation may be attributed to several interrelated mechanisms. Physiologically, high-altitude environments promote adaptive responses such as improved oxygen utilization, increased basal metabolic rate, and enhanced insulin sensitivity, which may counteract pollution-induced metabolic disruptions (30). Environmentally, lower atmospheric pressure and greater wind dispersion at high altitudes reduce pollutant concentration and exposure (31). Behaviorally, residents in elevated regions are more likely to engage in outdoor physical activities due to traditional lifestyles and less urban congestion, thereby mitigating sedentary behaviors commonly linked to urban air pollution (32). These factors jointly buffer the obesogenic effects of air pollutants in high-altitude settings.

PA and SQ mediated the association between air pollution and BMI. This finding underscores the complex interaction between environmental factors and lifestyle behaviors in influencing obesity risk. Mechanism analyses revealed that a chain mediator, consisting of PA and SQ sequentially, partially mediated this association. Previous studies identified PA and SQ as two separate mediators in the link between environmental exposures and BMI (20, 34). These studies have shown that both PA and SQ independently contribute to mitigating the adverse effects of environmental factors on metabolic health. However, our study is the first to reveal that PA and SQ could work as a chain mediator, simultaneously influencing the relationship between air pollution and BMI. This means that improving one factor, such as increasing physical activity, could have a ripple effect on sleep quality, which in turn further mitigates the impact of air pollution on BMI. This chain mediation of PA and SQ highlights the importance of promoting both physical activity and ensuring adequate sleep to mitigate the influence of air pollution on OW/OB in large-scale populations. Public health interventions that focus on improving both PA and SQ may be particularly effective in reducing obesity risk in populations exposed to high levels of air pollution.

### 4.4 Contributions and health recommendations

This study contributes to the literature by providing the first large-scale empirical evidence on the joint effects of air pollution and altitudes on OW/OB in China, emphasizing the need to consider co-exposure rather than isolated environmental factors. Using Bayesian Kernel Machine Regression and a moderated chain mediation model, the research demonstrates that altitudes significantly moderate the obesogenic impact of air pollution, offering a refined understanding of spatial heterogeneity in environmental health risks. Practically, the findings highlight the necessity for region-specific public health strategies: lower-altitude, high-pollution areas may require intensified environmental and behavioral interventions, while high-altitude regions exhibit physiological and behavioral resilience that could inform more targeted resource allocation. These insights advance both theoretical and applied frameworks in environmental epidemiology and precision public health.

Based on our findings, we recommend the following public health interventions for China. First, enhance air quality regulation: Implement stricter air quality controls to reduce exposure to PM_2.5_, BC, NMVOC, and CH_4_, which are linked to higher BMI, particularly in industrial and high-traffic areas. Second, promote behavioral interventions: Encourage physical activity and improve sleep quality to mitigate the effects of air pollution and altitude on BMI, particularly in high-pollution areas. Third, address socioeconomic disparities: Target interventions in medium-altitude regions to improve access to healthy food, physical activity, and healthcare, reducing obesity-related health disparities.

### 4.5 Limitations

This study has some limitations. Self–reported information may lead to downward bias in estimates. The BMI information of the Chinese individuals in this study was derived from self–reported height and weight, which may have resulted in the underestimation of BMI. This bias is acceptable given the widespread use of self–reported data in obesity analyses (20, 33). To address this, future studies should utilize specialized equipment, such as Dual-Energy X-ray Absorptiometry (DXA), to more accurately measure OW/OB. Second, only cross-sectional relationships were captured. To overcome this limitation, future cohort studies that examine the long-term effects of multiple pollutants on obesity in Chinese adults are needed. Additionally, some CFPS cohort samples from specific prefectures were excluded to maintain exposure diversity from geographic variation. Future studies should aim to include a broader, more geographically diverse sample to improve generalizability. Caution is required when interpreting the positive effects of CH_4_ and NMVOCs on obesity. Our analysis is based on population-level statistical data, and more detailed clinical randomized controlled trials are needed for further validation. Additionally, the selection of air pollution exposures was driven by data availability, and thus some important exposures could not be included in the analysis due to the unavailability of data.

## 5 Conclusion

This study examined the association of mixed exposure to air pollution and altitudes with OW/OB. Four air pollution exposure parameters were positively associated with OW/OB, altitude was negatively–positive–negatively related to BMI across concentration tertiles. Altitudes negatively moderated the relationship between air pollution and BMI, and a chain mediator, consisting of PA and SQ sequentially, partially mediated this association. Future studies are needed to focus more on multiple environmental exposures correlated with OW/OB.

## Data Availability

The original contributions presented in the study are included in the article/supplementary material, further inquiries can be directed to the corresponding author.
